# Effect of novel patient interaction on students’ performance of pregnancy options counseling

**DOI:** 10.3402/meo.v20.29401

**Published:** 2015-12-09

**Authors:** Angela Shaddeau, Abigail Nimz, Jeanelle Sheeder, Kristina Tocce

**Affiliations:** 1 Walter Reed National Military Medical Center, Bethesda, MD, USA; 2 Department of Obstetrics & Gynecology, University of Colorado Anschutz Medical Campus, Aurora, CO, USA

**Keywords:** options counseling, OSCE, abortion

## Abstract

**Background:**

Although options counseling is a fundamental skill for medical providers, previous research has identified gaps in medical school reproductive health education.

**Purpose:**

To determine if a 1-h novel patient interaction (NPI) improves student performance when caring for a standardized patient with an unintended pregnancy.

**Methods:**

From September 2012 to June 2013 we randomized third-year medical students at the University of Colorado School of Medicine to the standard curriculum plus an NPI, or the standard curriculum only. The NPI consisted of a 1-h small-group session with a patient who discussed her experiences with options counseling and her decision to terminate her pregnancy. Students completed an Objective Structured Clinical Examination (OSCE) at the rotation's end, which included options counseling. The primary outcome was the proportion of participants achieving ‘excellence’ on the OSCE checklist. ‘Excellence’ was defined as a score ≥90%. Examinations were flagged as ‘unsatisfactory encounters’ if core competencies were not addressed. OSCE standardized patients and evaluators were blinded to group assignment.

**Results:**

In total, 135 students were eligible and randomized: 75 to NPI; 60 to control. During the OSCE, few students achieved ‘excellence’ (24% NPI vs. 28% control, *p*=0.57).

There were no differences between scores for components of options counseling. More students in the control group ‘appeared somewhat uncomfortable’ delivering the pregnancy test results (5% NPI vs. 18% control, *p*=0.006). More than half (54%) of the intervention group and 67% of controls had ‘unsatisfactory encounters’ (*p*=0.16), almost exclusively due to omission of adoption. Most students addressed abortion (96% NPI vs. 92% control, *p*=0.29).

**Conclusions:**

A 1-h NPI does not improve medical students’ performance of pregnancy options counseling and the option of adoption is routinely omitted. Adoption is clearly an area that needs greater attention when designing comprehensive reproductive health curriculum for medical students.

Approximately half of the 6.6 million pregnancies in the United States each year are unintended (1), making options counseling a key component of reproductive health communication skills. The Association of Professors of Gynecology and Obstetrics (APGO) specifically addresses options counseling and abortion in the APGO Medical Student Educational Objectives: ‘Pregnancy termination is a reproductive option. Regardless of personal views about abortion, students should be knowledgeable about its public health importance as well as techniques and complications. The student should be able to provide non-directive counseling to patients surrounding pregnancy options’ (2).

Previous research has brought attention to gaps and barriers that are present in family planning medical student education, including the lack of training in standard curricula and few clinical opportunities for students (3–8). In a national survey assessing abortion training for medical students, 23% of clerkship directors stated that abortion was not covered in the third-year curriculum, and the majority of directors cited a lecture on abortion as the only consistent exposure to the topic during clinical training (6). Although there is a paucity of instruction, studies have shown that the majority of students have positive attitudes towards family planning education and desire clinical experiences in abortion care (3, 6, 9). Unfortunately, students are often unable to obtain these clinical exposures (9) and alternative methods of education have been explored.

Workshops on conscientious refusal have been shown to improve students’ communication skills, but not their performance of options counseling (10, 11). In order to provide all students with an opportunity to interact with women who have faced an unintended pregnancy, regardless of their clinical exposure during the clerkship, we designed a novel patient interaction (NPI). During the NPI, small groups of students interacted with a patient-facilitator who had undergone options counseling and pregnancy termination. Although some medical schools incorporate small-group sessions on abortion into their curriculum, this session incorporated real patients and allowed students to understand the clinical aspects of options counseling and abortion care from the patient's perspective. This unique experience could potentially broaden student perspectives and help students empathize with future patients.

We hypothesized that exposure to the NPI at the beginning of the clerkship would enhance students’ competence and comfort with options counseling, which we evaluated through an end-of-clerkship Objective Structured Clinical Examination (OSCE).

## Materials and methods

We received exempt status by the Colorado Multiple Instructional Review Board after this study was determined to pose minimal risk to subjects as defined by federal regulations, to occur in an established educational setting, and to compare instructional techniques/curricula. Each block of third-year medical students rotating through the Women's Care clerkship from September 2012 to June 2013 was randomized by a course administrator into one of the two groups (NPI or control). All third-year students enrolled in the Women's Care clerkship during this time were included in this study. At the clerkship orientation, it was explained to the NPI group that participation in this exercise was a compulsory but ungraded course element. All students were informed that the OSCE was also compulsory, but would not have an impact their clerkship grade.

The NPI group was exposed to both the standard curriculum and the NPI. The control group received only the standard third-year clerkship curriculum. During the study period, the family planning curriculum included in the clerkship consisted of two didactic sessions (contraception and abortion), a small-group ethics session (focused on conscientious refusal and legal aspects of abortion), and a simulation session (hands-on experience with manual vacuum uterine aspiration and intrauterine device insertion). Focused instruction on options counseling was not included in the standard curriculum.

The NPI consisted of small groups of students (≤5) spending 1 h with a patient-facilitator who volunteered to discuss her experience with options counseling, her decision-making process, and the termination of her pregnancy. Students then had the opportunity to ask the patient-facilitator questions about any aspect of her experience. Clerkship directors and staff were not present during the sessions in order to allow free conversation and questions between the patient-facilitator and the students. Immediately following the sessions, the patient-facilitators recorded what questions were asked of them and their overall impressions of the groups.

We required the patient-facilitators to have had a recent experience with options counseling, to be willing to discuss their personal experiences with small groups of medical students, and to participate in a 2-h training session. Our family planning clinic serves as a training facility for medical students, obstetrics/gynecology residents, and family planning fellows. The patient-facilitators each expressed interest in our educational model during their clinic visit. They were then invited by the Women's Care course director to become patient-facilitators.

The Women's Care course director and a research assistant conducted a 2-h training session. During this session, the patient-facilitators were instructed to cover core topics (how options counseling was performed, what was covered, what was done well, what could have been improved, how they ultimately decided to terminate their pregnancy, what their experience was with the procedure and recovery, and finally, what they desired from their providers to make their experience better). They were also given information on what should ideally be included during all options counseling sessions in the clinical setting. Each patient-facilitator agreed to an open question session after the discussion of core topics. Each patient-facilitator led an equal number of sessions during the study.

At the end of each block, all students participated in an options counseling OSCE. This OSCE was designed at the Miller School of Medicine, University of Miami, and had been piloted on 105 students. Permission was obtained from Dr. Carla S. Lupi to use the OSCE, which included a standardized checklist that assessed communication skills and proficiency with options counseling (12, 13). Students in this study participated in the OSCE at the Center for Advancing Professional Excellence (CAPE), at the University of Colorado School of Medicine. Through the CAPE's educational staff, the standardized patients were each trained for participation in this scenario and evaluation of the students with a checklist.

In the OSCE, the setting is described as an urgent care and the student is given ‘sign-out’ consisting of a focused history and physical examination for a patient with vague gastrointestinal complaints, fatigue, a slightly enlarged uterus, and a positive pregnancy test. The student is charged with informing the patient of her positive pregnancy test and completing the encounter. The patient does not suspect that she is pregnant and responds to the student with emotional silence and deep uncertainty at the news of the pregnancy. The patient then asks the student two ethically challenging questions: ‘What would you do?’ and ‘What should I do?’

Immediately following the encounter, the standardized patients completed a checklist. Checklists were numerically scored. They were also flagged as ‘unsatisfactory encounters’ if the student did not accomplish the following core competencies: neutrally inform patient of the positive pregnancy test; acknowledge the patient's distress; ask her how she feels about the pregnancy; show interest and openness to the patient's concerns/beliefs; respond to the patient's questions without interjecting his/her own perspectives/values; and present parenting, abortion, and adoption as options. The standardized patients were blinded to students’ group assignments.

In a post-exercise questionnaire, each student assessed his/her self-perceived competence and comfort level with the patient encounter. They were also asked questions regarding their clinical experience with disclosure of pregnancy test results and/or options counseling, basic demographics, and religiosity.

Descriptive statistics were calculated as means and proportions. Chi-square and Fisher's exact tests were used to compare dichotomous and categorical variables; Student's *t*-tests were used to compare continuous variables. Data analysis was carried out with blinded group assignment. IBM SPSS statistical software (version 22.0.0) was used for all analyses. The primary outcome was the proportion of participants achieving ‘excellence’ on the OSCE checklist. Secondary outcomes included student performance of specific components of options counseling, standardized patient-perceived comfort with options counseling, student-perceived performance, and impressions of the NPI patient-facilitators.

‘Excellence’ was defined as a score of 90% or above on the checklist (the score that an options counselor in our clinic routinely achieves). We estimated that half of the students would demonstrate ‘excellence’ in options counseling with the standard clerkship curriculum. With exposure to the NPI, we projected that 80% would demonstrate ‘excellence’. Based on these predictions, approximately 45 students would be needed in each group to show a significant difference with a type-1 error of 0.05 and a power of 0.8. We estimated that at least 10% of the students would not be available for participation, so our actual samples were larger than 45 in each group.

## Results

**Fig. 1:**
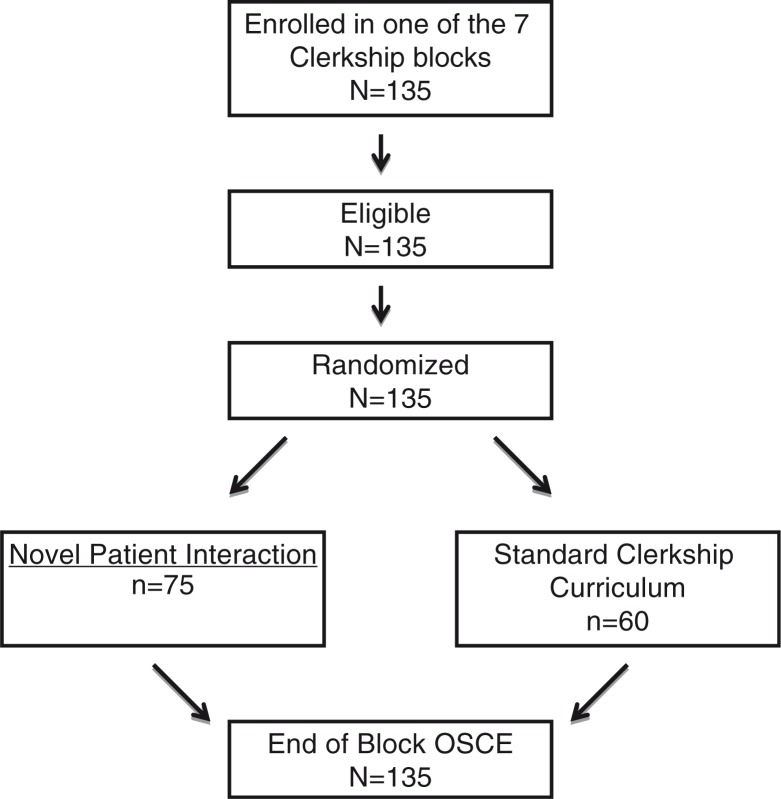
Participant flow.

All third-year students rotating through the Women's Care clerkship during the study period were eligible for the study and participated. Over the course of seven clinical blocks there were 135 students; 75 were randomized to the intervention group and 60 to the control group. Of the 75 students in the intervention group, each participated in the NPI. All 135 students completed the OSCE on the last day of their clerkship (Fig. 1).

The NPI and control groups were demographically similar regarding sex, age, and religiosity (Table 1). The intervention group had a significantly higher percentage of Caucasian students compared to the controls (80% vs. 67%, *p*=0.04). Of the entire cohort, 41% reported the opportunity to perform options counseling during their clerkship; 16% indicated they obtained experience during other rotations.

**Table 1:** Baseline characteristics of participants

Characteristic	Intervention group *n=*75 *n* (%)	Control group *n=*60 *n* (%)	*p*
Sex			0.17
Female	35 (46.7)	29 (48.3)	
Age group (years)			0.28
20–25	17 (22.7)	16 (26.7)	
26–30	39 (52)	34 (56.7)	
31–35	14 (18.7)	8 (13.3)	
36–40	2 (3.3)	1 (1.3)	
Over 40	0 (0)	1 (1.3)	
Race/ethnicity			0.04*
Caucasian/white	50 (66.7)	48 (80)	
Hispanic/Latino	6 (8)	2 (3.3)	
Black	2 (2.7)	1 (1.7)	
Asian	4 (5.3)	7 (11.7)	
Other	10 (13.3)	2 (3.3)	
Religious affiliation			0.08
Catholic	15 (20)	8 (13.3)	
Hindu	0 (0)	2 (3.3)	
Jewish	5 (6.7)	2 (3.3)	
Muslim	1 (1.3)	2 (3.3)	
Other Christian faith	21 (28)	10 (16.7)	
No religious affiliation	22 (29.3)	31 (51.7)	
Other	8 (10.7)	4 (6.7)	
Relationship status			0.11
Single	26 (34.7)	17 (28.3)	
Married	21 (28)	14 (23.3)	
In a relationship	23 (30.7)	19 (31.7)	
Living with significant other	2 (2.7)	9 (15)	

* *p*<0.05.

Few students in either group achieved ‘excellence’ in options counseling (≥90%): 24% of the NPI group vs. 28% of controls (*p*=0.57). The entire cohort achieved a mean score of 84%±11.0%; the NPI group mean was 84%±11.0% vs. 83%±10% in the controls (*p*=0.82). Despite these high mean scores, more than half of the checklists were flagged as overall ‘unsatisfactory performances’, almost exclusively due to omission of adoption as an option. Of the entire cohort 40% mentioned adoption, and there was no difference by group (Table 2).

**Table 2:** Student performance of options counseling

OSCE performance (*N*=135)	Intervention *n* (%)	Control *n* (%)	*p*
Mean score	84%	83%	0.08
‘Excellence’ (90%+score on checklist items)	18 (24)	17 (28.3)	0.57
Flagged as ‘unsatisfactory performance’	41 (54.7)	40 (66.7)	0.16
Competency performance			
Introduced self	75 (100)	58 (96.7)	0.20*
Asked about contraception use	54 (72)	44 (73.3)	0.86
UPT results delivered in a neutral fashion	74 (98.7)	60 (100)	1.0*
Established trust with the patient	63 (84)	54 (90)	0.31
Appeared somewhat uncomfortable when delivering pregnancy test results	4 (5)	11 (18)	0.006
Acknowledged the following options			
Continuation	71 (94.7)	56 (93.3)	0.75*
Abortion	72 (96)	55 (91.7)	0.29*
Adoption	35 (46.7)	20 (33.3)	0.12

* Fisher's exact test.

The failure to acknowledge adoption as an option was a clear outlier. Almost all students addressed continuation of pregnancy (95% of NPI participants and 93% of controls, *p*=0.75) and abortion during the OSCE (96% of NPI participants and 92% of controls, *p*=0.29). Significantly more students offered parenting as a pregnancy option compared to adoption in each group (NPI: 95% vs. 47%, *p<*0.001; controls: 92% vs. 33%, *p<*0.001). Students also were more apt to discuss the option of abortion compared to adoption in each group (NPI: 96% vs. 47%, *p*<0.001; controls: 93% vs. 33%, *p<*0.001).

Although few students achieved ‘excellence’, students’ performance of the major communication skills during the OSCE was strong. Almost universally, they presented the pregnancy test results in a neutral fashion (99% of the NPI group and 100% of the control group, *p=*0.27). Most asked how the patient felt about the pregnancy (83% of the NPI group and 88% of the control group, *p=*0.48) and were able to ‘establish trust’ with her (84% of the NPI group and 90% of controls, *p=*0.39). Even without the NPI, the students’ handling of the ethically challenging questions was strong; ≥84% of each group answered the standardized patient's questions without revealing their own personal beliefs and appeared non-judgmental while doing so.

Immediately following the OSCE, many students recognized that they were not complete in their options counseling (49% of the NPI group and 41% of the controls, *p*=0.32). Few students reported ethical/moral conflict during the exercise (14% of the NPI group vs. 17% of controls, *p*=0.63) and almost all students felt that they completed their counseling in a non-judgmental fashion (99% of NPI vs. 97% of controls, *p*=0.44). Increased comfort with options counseling as a result of the NPI was affirmed in 61% of students in that group. A similar percentage of controls (62%) stated that an NPI would have increased their comfort level while performing options counseling. The standardized patients classified a greater proportion of students in the control group as appearing ‘somewhat uncomfortable’ with delivering pregnancy test results than the NPI group (5% NPI vs. 18% control, *p*=0.006).

Additional analyses of the achievement of ‘excellence’, the ‘unsatisfactory performances’, and the completion of specific portions of options counseling between both groups showed no significant association with any demographic variables, religiosity, or clerkship sites. When students assigned to the Catholic clerkship site (*n*=31) were compared with those at secular institutions (*n*=104), equal percentages achieved ‘excellence’ (26%). ‘Unsatisfactory performances’ were also similar (71% vs. 56%, *p*=0.15).

Last, the questions and impressions recorded by the patient-facilitators were reviewed. The patient-facilitators, who were Hispanic and in their early 20s, reported that the most common questions asked by the students focused on support from the father of the pregnancy, physical recovery, what she would do if she could not access abortion, what could have been done to prevent her unintended pregnancy, and her emotions post-procedure. The patient-facilitators’ impressions of the student groups were overall positive. Students were thankful, respectful, and sought advice on addressing their future patients. A few students were simply ‘not interested’ or spoke in a manner that made the patient-facilitators uncomfortable. Some were noted to have cried during the session and expressed that this small group ‘changed their entire outlook’ regarding reproductive options. Numerous students recommended that the patient-facilitators write a book to share their experiences with more students and patients.

## Discussion

This randomized trial of the effect of participating in an NPI on options counseling did not significantly improved students’ performance of pregnancy options counseling as measured by an end-of-rotation OSCE. We did not anticipate that so few students would achieve ‘excellence’ after the NPI, or that a sizeable percentage of checklists in both groups would be classified as ‘unsatisfactory performances’ due to the exclusion of one core objective: failure to present adoption as a pregnancy option.

We hypothesized that the gaps in student competency with options counseling would lie in presenting abortion as an option. However, students almost universally discussed abortion as an option during the OSCE. In addition, the standardized patient evaluations indicate that almost every student handled this discussion in a non-judgmental way. Adoption was the unexpected outlier, with only 41% of the entire cohort mentioning this option to the standardized patient. Lupi et al. (12) reported similar findings regarding the disregard of adoption. Perhaps this is due to abortion being specifically presented as part of the clerkship curriculum, where adoption is not. Previous research has focused on the importance of incorporating abortion care into medical school curriculum (6–9), whereas there is no such attention on standardization of adoption curriculum.

Students may also have heightened awareness of abortion due to political debate and media attention (8). Without similar debates surrounding adoption, students may not have any exposure to adoption outside of the educational setting. Adoptions in the United States have also declined; the estimated effect of abortion legalization on adoption rates is sizable and can account for much of this decline (14). Without formal integration of adoption into the curriculum, medical students may continue to overlook this option when careing for patients with unintended pregnancies.

Although the NPI group did not show improved performance with options counseling, impressions from the patient-facilitators indicated that the large majority of students were very engaged during the sessions and expressed gratitude for the experience. Those who participated in the NPI were perceived by the standardized patient to be more comfortable with delivering the positive pregnancy test result and handling the patient's uncertainty than those who did not participate in the NPI. Improved comfort may ultimately translate into better care for patients facing challenging reproductive decisions.

Meeting the APGO objectives for competence in non-directive pregnancy options counseling will require comprehensive curricular emphasis on all three options: parenting, abortion, and adoption. However, many medical schools do not include any standardized family curriculum or clinical experience in undergraduate training (6). It is unlikely that all clerkships will be able to provide and/or require clinical experience, as residency programs do. Didactics, workshops, small-group sessions, and simulations will be necessary. Clearly, the optimal combination of these modalities has yet to be defined. Based on our study results and those of Lupi et al. (12), as more research is performed to define the ideal curriculum, focused discussion of adoption and more explicit review of the individual components of pregnancy options counseling should be incorporated into any future interventions.

Strengths of this study include the randomized design with assessments performed by blinded evaluators, use of a validated OSCE and assessment tool (12), and participation of all eligible students during the study period. Block randomization decreased the chances of the NPI students discussing the exercise with control students prior to their OSCE assessment exercise. Although we enrolled more students than our sample size calculation, our estimates on how many students would achieve ‘excellence’ was higher than our actual results (we assumed 50% of controls would achieve ‘excellence’ and only 28% did). Although not statistically significant, the rate of ‘excellent’ scores was actually lower among the NPI. A larger cohort would likely establish bounds on what the effect of the NPI would be on ‘excellent’ scores; but at present, there was no discernible effect.

The NPI was designed to increase student empathy for patients undergoing challenging decisions regarding pregnancy and improve their competence in options counseling by recognizing what patients’ desire from providers during this process. The lack of authority figure presence during the sessions was purposeful, with the intent of encouraging students’ questions and free-flowing conversation. Although patient-facilitators were trained to cover the core objectives, particular students could theoretically have derailed sessions. Also, because two different NPI facilitators were used, there were also possible differences in the NPI experience. Other weaknesses include the underrepresentation of certain demographic groups at the University of Colorado School of Medicine. Compared to the 2014 overall US medical student demographics (15), this sample had more Caucasian (73% compared with 57%) and Hispanic/Latino students (6% vs. 4%), but fewer Asian (8% vs. 20%) and African-American (2% vs. 6%) students. In addition, the trained standardized patients, not physicians, performed the OSCE assessments.

Although the NPI did not improve students’ performance of options counseling, we have undoubtedly identified adoption as area that needs greater attention when designing comprehensive reproductive health curriculum for medical students. Standardized patients perceived the NPI group to be more comfortable when handling complex clinical situations, which has important implications for improving patient comfort and satisfaction. Future research is needed to determine the optimal combination of educational activities and clinical exposures to ensure medical student competency with options counseling.
